# A Pilot Study Investigating the Use of Regional Oxygen Saturation as a Predictor of Ischemic Wound Healing Outcome after Endovascular Treatment in Patients with Chronic Limb-Threatening Ischemia

**DOI:** 10.3400/avd.oa.20-00132

**Published:** 2021-03-25

**Authors:** Takafumi Kayama, Masaki Sano, Kazunori Inuzuka, Kazuto Katahashi, Tatsuro Yata, Yuta Yamanaka, Ena Naruse, Naoto Yamamoto, Hiroya Takeuchi, Naoki Unno

**Affiliations:** 1 Division of Vascular Surgery, Hamamatsu University School of Medicine, Hamamatsu, Shizuoka, Japan; 2 Second Department of Surgery, Hamamatsu University School of Medicine, Hamamatsu, Shizuoka, Japan; 3 Division of Vascular Surgery, Hamamatsu Medical Center, Hamamatsu, Shizuoka, Japan

**Keywords:** pilot study, oximetry, near-infrared spectroscopy, ischemia, wound healing

## Abstract

**Objective**: To determine the prognostic value of regional tissue oxygenation saturation (rSO_2_) for ulcer healing after endovascular treatment (EVT) of peripheral arterial disease (PAD).

**Materials and Methods**: Among PAD patients, 34 patients with chronic limb-threatening ischemia underwent EVT for limb salvage. We retrospectively analyzed the cutoff rSO_2_ values on postoperative day 1 to predict ulcer healing and patient prognosis. Skin perfusion pressure (SPP) and transcutaneous oxygen pressure (TcPO_2_) were also used to assess wound healing.

**Results**: A finger-mounted tissue oximeter can easily measure rSO_2_ on the dorsal foot. Among the 34 patients, the ulcer healed in 25, and no changes were observed in 2 patients at 1 month after EVT. However, 7 patients needed major amputation at the same time. Wound healing was achieved in all patients with rSO_2_≥50%. With this cutoff, the sensitivity and specificity of the new device for wound healing were 100% and 64%, respectively. In all the wound healing cases, SPP was ≥45 mmHg, and TcPO_2_ was ≥40 mmHg.

**Conclusion**: To assess limb ischemia, rSO_2_ can be measured quickly and easily using this device. We suggest that an rSO_2_>50% shows good prognosis for ulcer healing.

## Introduction

The prevalence of peripheral arterial disease (PAD) has been reported to be 3%–10%, increasing to 15%–20% in people aged >70 years, and 0.1% of the population experience chronic limb-threatening ischemia (CLTI).^1–3)^ In Japan, the prevalence of PAD has been reported to be 1%–3%, and the incidence is increasing.^4,5)^ CLTI represents the most advanced form of PAD and is associated with high rates of mortality and amputation.^1)^ Several guidelines on PAD have been published. The Society for Vascular Surgery Wound, Ischemia and foot Infection (WIfI) classification system has been proposed to predict 1-year amputation risk and potential benefits from revascularization.^6)^ Assessing the blood flow of lower limbs is important in this context.

Various non-invasive methods have been used to diagnose PAD, including ankle brachial index (ABI), toe brachial index (TBI), skin perfusion pressure (SPP),^7)^ and transcutaneous oxygen pressure (TcPO_2_). However, these methods have some limitations. ABI is the current gold standard for evaluating PAD worldwide, and values≤0.9 have a specificity of ≥98% to detect PAD. However, ABI values above 1.3 are commonly observed in patients with diabetes or chronic kidney disease, probably due to the calcification of arteries of the lower extremities,^8)^ such that false-negative ABI results occur in 17%–24% of patients with diabetes and on hemodialysis.^9)^ ABI measurements are painful for patients because of the compression exerted by the cuff. Moreover, the measurement site is limited to the ankle.^10)^ The American College of Cardiology Foundation/American Heart Association 2005 guidelines similarly defined TBI<0.7 as a diagnostic for PAD; however, none of the studies cited in support of this cutoff value actually advocated the use of TBI<0.7 as a diagnostic tool. Moreover, TBI can diagnose PAD, but fails to give an indication of its severity.^11)^ SPP is a non-invasive method that uses laser Doppler measurement to measure arterial microcirculatory pressure at the skin level and slowly reduces the inflation cuff pressure at the measurement site to detect red blood cell movement. Although SPP is believed to help assess the severity of lower limb ischemia, patients often experience intolerable pain caused by cuff pressure.^7)^

TcPO_2_ was developed to measure skin tissue oxygenation. When carbon dioxide (PCO_2_) is released from the tissue, it diffuses through the membrane into the electrolyte, where it reacts with carbonated water. The signal detected at the electrodes is digitized and reconverted to pressure of oxygen (PO_2_) and PCO_2_ expressed in mmHg. Measuring TcPO_2_ requires a warm-up period of 15 min for the sensor electrodes, a flat skin surface on which to place the sensors, and a stable body position.^12,13)^

It is difficult to use the traditional diagnostic methods in patients with rest pain and skin ulcers. Therefore, we focused on a novel, quick, simple, accurate, and painless method that can be used on any skin area; near-infrared spectroscopy (NIRS) is used to assess tissue oxygenation. In NIRS, the light-emitting diodes emit light at two different wavelengths, which penetrate the tissue. The reflection wavelengths obtained from oxygenated hemoglobin and deoxygenated hemoglobin are used to calculate the regional tissue oxygenation saturation (rSO_2_).^13,14)^ The method was developed more than 20 years ago and has been used for limb blood flow evaluation in muscles with a depth of 30 mm or more; it is useful in PAD patients with intermittent claudication. However, its usefulness in skin blood flow and wound healing has not been shown.^15,16)^ Although available, these previously described tests are unsuitable for predicting prognosis after postoperative revascularization. Currently, no test can be used as an index for improving blood circulation in the early postoperative period of endovascular treatment (EVT).

A ﬁnger-mounted tissue oximeter using the NIRS technique (Toccare: Astem Co., Ltd., Kawasaki, Japan) was developed, enabling skin measurements at a 5-mm depth.^14)^ In obstetrics and gynecology, this device is used transvaginally to touch the scalp of the fetus and evaluate fetal acidosis.^17,18)^ Assessment of lower limb blood flow using this device has already been performed in patients with PAD and in healthy volunteers, and its correlation with conventional devices has been shown.^19)^ In this study, a newly developed ﬁnger-mounted tissue oximeter was used to measure tissue oxygenation before and after EVT, with the purpose of assessing lower limb blood flow to predict wound healing and treatment efficacy.

## Materials and Methods

### Participants

**Figure 1:**
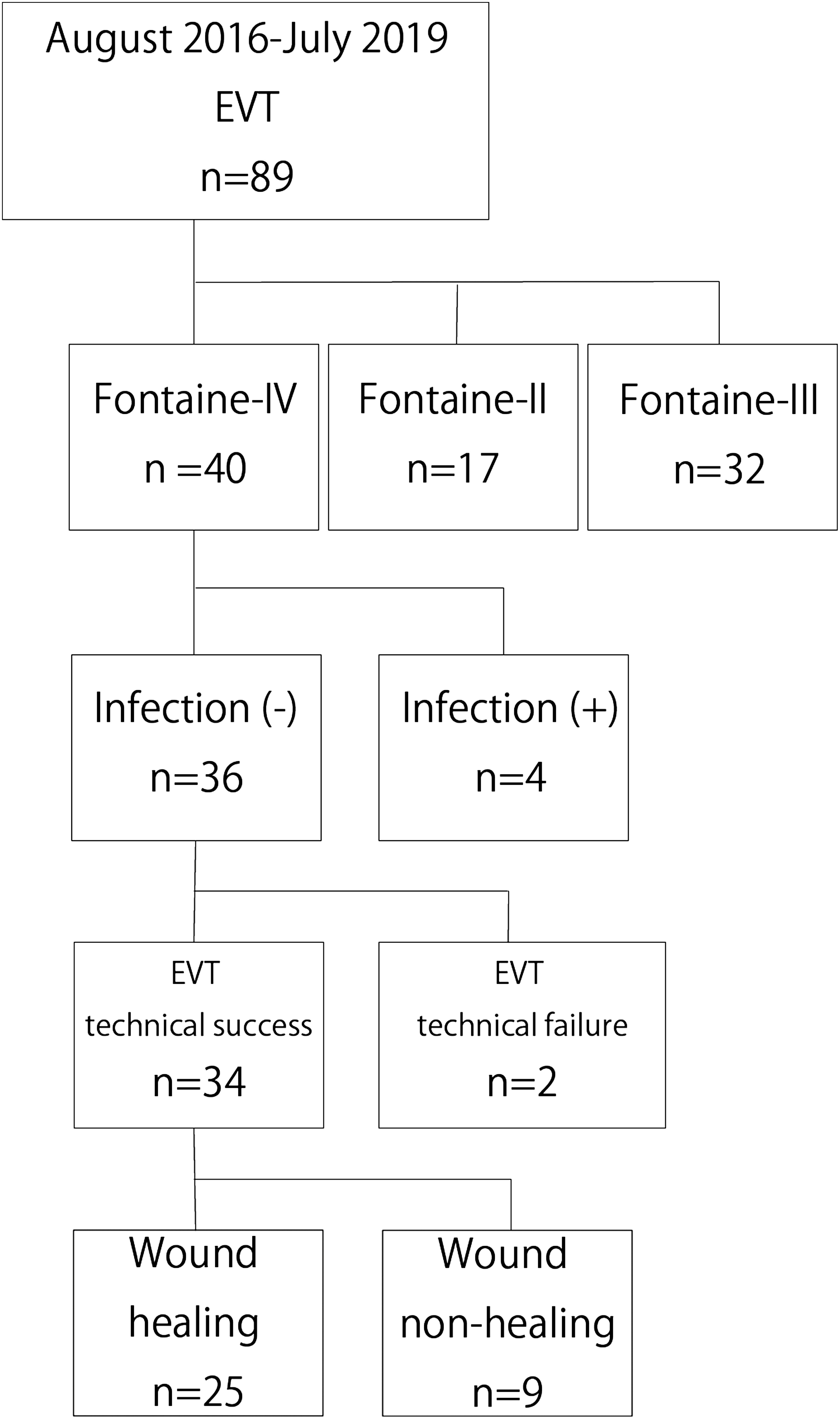
Fig. 1 Patient selection and classification scheme.

In this single-center observational study, we retrospectively analyzed the database of patients with CLTI who underwent EVT in the Hamamatsu University School of Medicine Hospital from January 2016 to April 2019 and consented to follow-up. When treating patients with CLTI, we first assess whether bypass surgery is possible or not based on the patient’s condition, and if the patient’s condition is poor, we consider EVT. All patients had non-healing ulcerations or gangrene (Fontaine stage IV). Lower limb echo-color Doppler was performed preoperatively on all patients to confirm the absence of varicose veins or deep vein thrombosis in order to exclude venous ulcers. Patients were included in the study if they had successful revascularization of at least one artery among the iliac, superficial femoral, popliteal, tibioperoneal trunk, anterior tibial, posterior tibial, or peroneal arteries at the final EVT angiography. Patients with rest pain but no ulceration or gangrene (Rutherford class 4), with EVT failure, and with infection based on the WIfI classification criteria for foot infection were excluded (**Fig. 1**).

EVT: endovascular treatment

Patients were divided into two groups: those who achieved wound healing without major amputation 1 month after surgery were assigned to the wound healing group, and those who did not were assigned to the non-wound healing group.

### Study approval

The study protocol was approved by the Ethical Review Board of Hamamatsu University School of Medicine (approval number 16-057). The study protocol was registered in the UMIN Clinical Trials Registry (UMIN-CTR; ID: UMIN000025021). Written informed consent was obtained from all participants.

### Procedure

A team of 2 vascular surgeons and 1 vascular technician performed the tests in our institution. Before EVT, we first measured rSO_2_ of the foot dorsum with the new device. Next, we measured TcPO_2_ of the foot dorsum, then ABI, and finally SPP. We allowed 5 min of rest between ABI and SPP measurements to eliminate the side effects of cuff pressure. On the first day after EVT, we measured rSO_2_, TcPO_2_, ABI, and SPP of the foot dorsum in the same manner.

The instruments used for each measurement were as follows: ABI: BP-203, Omron, Kyoto, Japan; SPP: SensiLase PAD 3000, Vasamed, Eden Prairie, MN, USA; TcPO_2_: TCM 4 series, Radiometer, Copenhagen, Denmark; and rSO_2_: Toccare: Astem Co., Ltd., Kawasaki, Japan. All procedures and measurements were performed with patients in the supine position. The temperature of the laboratory was maintained at 25°C.

All EVT procedures were performed with the patients under local anesthesia, and no oxygen was given to the patients before or after treatment. Our vascular surgery unit has a wound management team with plastic surgeons, rehabilitation doctors, and nutritionists, which manages the patients’ wounds after EVT.

### Clinical outcomes

The endpoint of this study was wound healing without major amputation (above or below the knee) or treatment re-intervention. All patients were followed up at 1 week, 1 month, and 3 months after treatment, with vascular surgeons determining the extent of wound healing.

### Statistical analysis

The unpaired t-test was used to compare continuous variables, and the chi-square test was used to compare categorical variables between the wound healing and non-healing groups. Data are shown as mean±standard deviation. To determine whether rSO_2_, TcPO_2_, and SPP measurements can function as predictors of wound healing, we calculated the threshold values for rSO_2_, TcPO_2_, and SPP for predicting wound healing in patients with CLTI. Receiver operating characteristic (ROC) analysis was performed. p values <0.05 were considered to be statistically significant. Data analyses were performed using SPSS 24.0 software (IBM Corporation, Armonk, NY, USA).

## Results

Between August 2016 and July 2019, 89 EVT procedures were performed in our department, of which 40 were performed for patients with Fontaine stage IV (**Fig. 1**). Four cases of foot infections and 2 cases with technical failure were excluded. The remaining 34 patients were included in this study. Patients’ demographics, stratified by groups, are shown in **Table 1**. Among the 9 wound non-healing patients, 4 underwent repeat EVT, and 2 underwent major amputation within 1 month after surgery. When background factors were compared between the two groups, there was a tendency for the wound healing group to be slightly depleted in dialyzed cases compared with the wound non-healing group, but this was not statistically significant. Next, the condition of the lower extremity was evaluated, but there were no significant differences between the wound healing group and the non-healing group in preoperative ABI, SPP, TcPO_2_, and rSO_2_ (**Table 1**). Regarding ulcer location, most ulcers were located on the toes, and patients with arterial lesions limited to the region below the knee tended to be more frequent in the non-healing group than in the wound healing group (**Table 1**).

**Table 1:** Table 1 Baseline characteristics of the patients and lower limbs

	All (n=34)	Wound healing (n=25)	Wound non-healing (n=9)	p Value
Age, years	74.7±9.7	74.2±9.8	76.3±9.2	0.572
Male sex	25 (73.5%)	18 (72.0%)	7 (77.8%)	0.917
Hypertension	18 (52.9%)	14 (56.0%)	4 (44.4%)	0.707
Diabetes mellitus	17 (50.0%)	13 (52.0%)	4 (44.4%)	0.719
Dyslipidemia	16 (47.1%)	12 (48.0%)	4 (44.4%)	0.837
Hemodialysis	20 (58.8%)	12 (48.0%)	8 (88.9%)	0.050
Smoking	23 (67.6%)	15 (60.0%)	8 (88.9%)	0.214
Coronary artery disease	22 (64.7%)	14 (56.0%)	8 (88.9%)	0.113
Cerebrovascular disease	5 (14.7%)	5 (20.0%)	0 (0%)	0.293
Total protein level, mg/dL	6.8±0.5	6.8±0.4	6.9±0.8	0.727
Albumin level, mg/dL	3.5±0.5	3.5±0.5	3.5±0.5	1.000
Hemoglobin level, mg/dL	10.7±1.9	10.7±2.0	10.8±1.4	1.000
HbA1c level, %	6.3±1.0	6.4±1.1	6.1±0.8	0.396
Total cholesterol level, mg/dL	157.2±30.1	154.7±31.9	164.1±25.6	0.390
Triglyceride level, mg/dL	103.8±42.7	100.0±37.5	114.9±52.1	0.447
Rutherford category 5	33 (97.1%)	24 (96.0%)	9 (100%)	
Rutherford category 6	1 (2.9%)	1 (4.0%)	0 (0%)	0.543
WIfI stage 1	5 (14.7%)	5 (20.0%)	0 (0%)	
WIfI stage 2	4 (11.8%)	4 (16.0%)	0 (0%)	
WIfI stage 3	18 (52.9%)	11 (44.0%)	7 (77.8%)	
WIfI stage 4	7 (20.6%)	5 (20.0%)	2 (22.2%)	0.111
Location of the ulcer				
Toe	28 (82.3%)	20 (80.0%)	8 (88.9%)	
Dorsum	3 (8.8%)	3 (12.0%)	0 (0%)	
Heel	2 (5.9%)	1 (4.0%)	1 (11.1%)	
Ankle	1 (2.9%)	1 (4.0%)	0 (0%)	
ABI before EVT	0.56±0.17 (n=31, 91.2%)	0.56±0.20 (n=23, 92.0%)	0.56±0.08 (n=8, 89.9%)	1.000
SPP before EVT	30.8±12.7 (n=24, 70.6%)	30.1±12.4 (n=18, 72.0%)	32.8±13.3 (n=6, 66.7%)	0.654
TcPO_2_ before EVT	23.4±20.6 (n=29, 85.3%)	24.1±21.1 (n=22, 88.0%)	21.3±18.7 (n=7, 77.8%)	0.756
rSO_2_ before EVT	46.3±4.5 (n=34, 100%)	46.5±4.5 (n=25, 100%)	45.9±4.4 (n=9, 100%)	0.732
Treated lesion				
Aortoiliac	5 (14.7%)	4 (16.0%)	1 (11.1%)	0.846
Femoropopliteal	23 (67.6%)	19 (68.0%)	4 (44.4%)	0.187
Infrapopliteal	16 (47.1%)	8 (32.0%)	8 (88.9%)	0.006
EVT procedure				
A-I	2 (5.9%)	2 (8.0%)	0 (0.0%)	0.382
A-I+Fem-Pop	2 (5.9%)	2 (8.0%)	0 (0.0%)	0.382
A-I+Fem-Pop+tibial	1 (2.9%)	0 (0.0%)	1 (11.1%)	0.091
Fem-Pop	14 (41.2%)	13 (52.0%)	1 (11.1%)	0.033
Fem-Pop+tibial	6 (17.6%)	4 (16.0%)	2 (22.2%)	0.675
Tibial	9 (26.25%)	4 (16.0%)	5 (55.6%)	0.021

Data are presented as mean±standard deviation or n (%). HbA1c: glycated hemoglobin; WIfI: Society for Vascular Surgery Wound, Ischemia and foot Infection; ABI: ankle brachial index; EVT: endovascular treatment; SPP: skin perfusion pressure; TcPO_2_: transcutaneous oxygen pressure; rSO_2_: regional tissue oxygenation saturation; A-I: iliac artery; Fem-Pop: superficial femoral artery and popliteal artery

With regard to the EVT procedures, there were 34 technically successful EVT procedures. The distributions of the focal arterial target lesions for revascularization are shown in **Table 1**. Comparison of the two groups identified that EVT of a solitary tibial artery was most likely to be associated with non-wound healing.

ABI, SPP, TcPO_2_, and rSO_2_ measurements were performed before and after EVT, but some patients could not be tested for ABI and SPP because of excessive pain caused by cuff pressure (3 and 5 patients for ABI, 10 and 8 for SPP, before and after surgery, respectively). The TcPO_2_ measurement requires at least 15 min of rest but could not be performed because of involuntary movements and pain in 5 and 6 patients before and after the operation, respectively. The rSO_2_ measurement was performed in all patients without difficulty (**Supplemental Table 1**).

**Figure 2:**
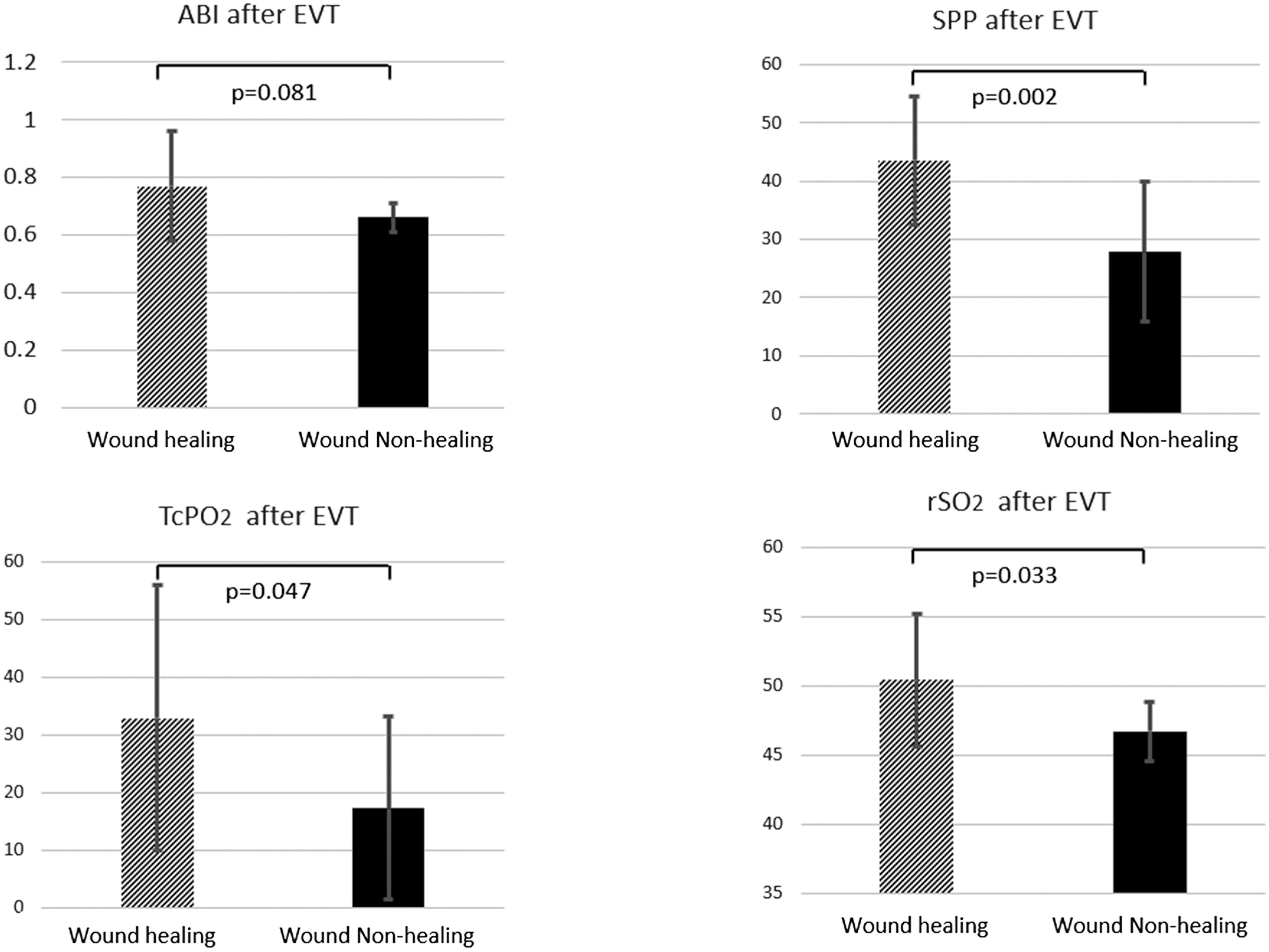
Fig. 2 Comparison of postoperative blood flow assessments between the wound healing and non-wound healing groups.

On postoperative day (POD) 1, ABI values were 0.77±0.19 in the wound healing group and 0.65±0.06 in the non-healing group. SPP values were 44.5±10.6 in the wound healing group and 27.9±12.1 in the non-healing group. TcPO_2_ values were 34.3±23.3 in the wound healing group and 14.6±15.0 in the non-healing group. rSO_2_ values were 50.1±4.8 in the healing group and 46.4±2.0 in the non-healing group (**Fig. 2**). ABI was elevated compared with the preoperative values, but without statistical significance. All the other tests demonstrated significant changes after EVT.

EVT: endovascular treatment

**Figure 3:**
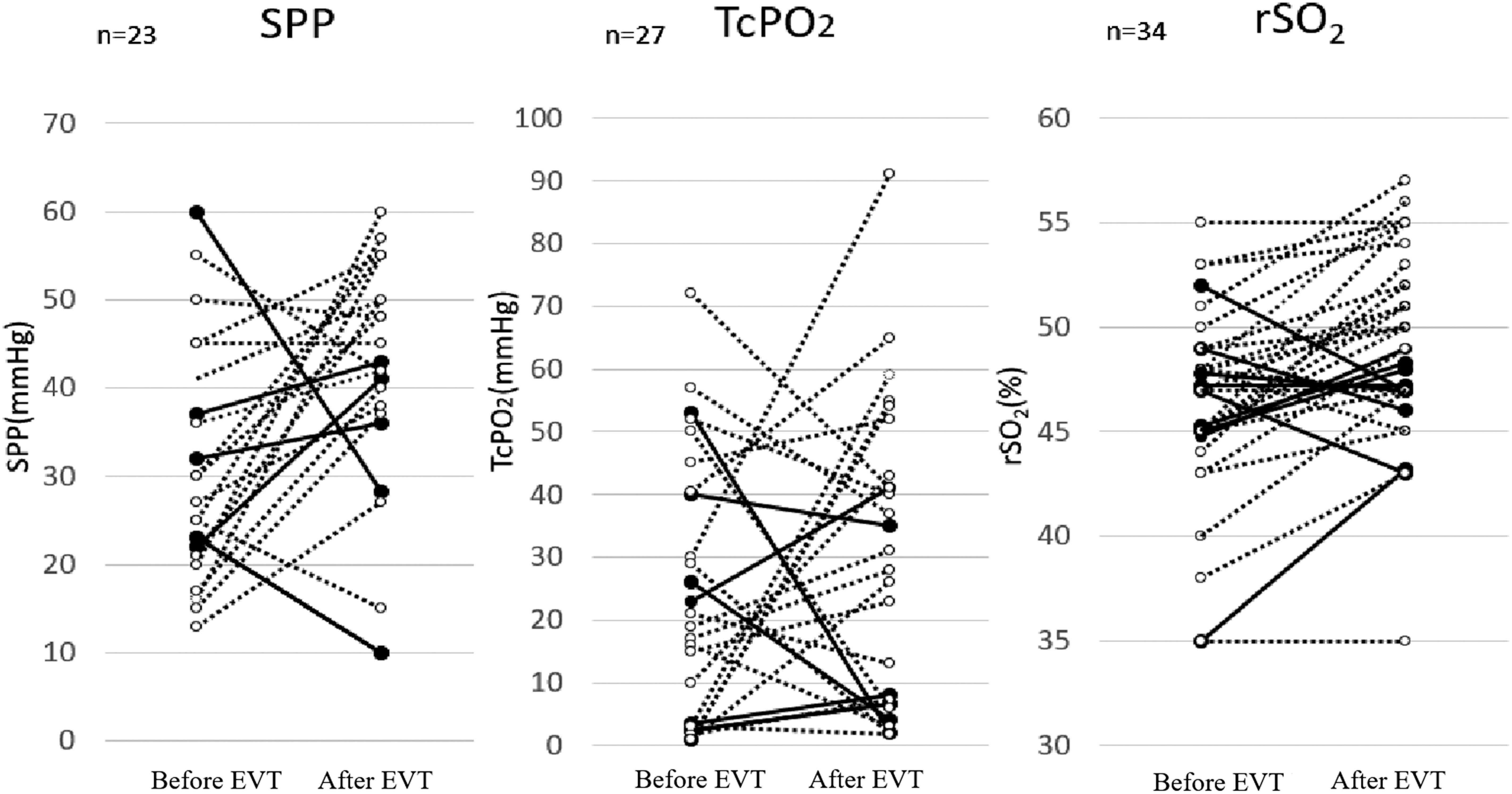
Fig. 3 Changes in blood flow measurements before and after EVT. Each line represents a change in the value for each case. Dotted and solid lines represent the wound healing group and the non-wound healing group, respectively.

EVT: endovascular treatment; SPP: skin perfusion pressure; TcPO_2_: transcutaneous oxygen pressure; rSO_2_: regional tissue oxygenation saturation

**Figure 3** shows the transition of perfusion parameters of SPP, TcPO_2_, and rSO_2_ from pre-EVT to POD 1 in each case. Some cases had values that worsened even after successful EVT procedures, and they were associated with wound non-healing. There were cases of wound non-healing even though the patients’ SPP or TcPO_2_ values were >40 mmHg. However, wound healing was achieved in all patients with rSO_2_ values of 50% or more after EVT (**Fig. 3**).

**Figure 4:**
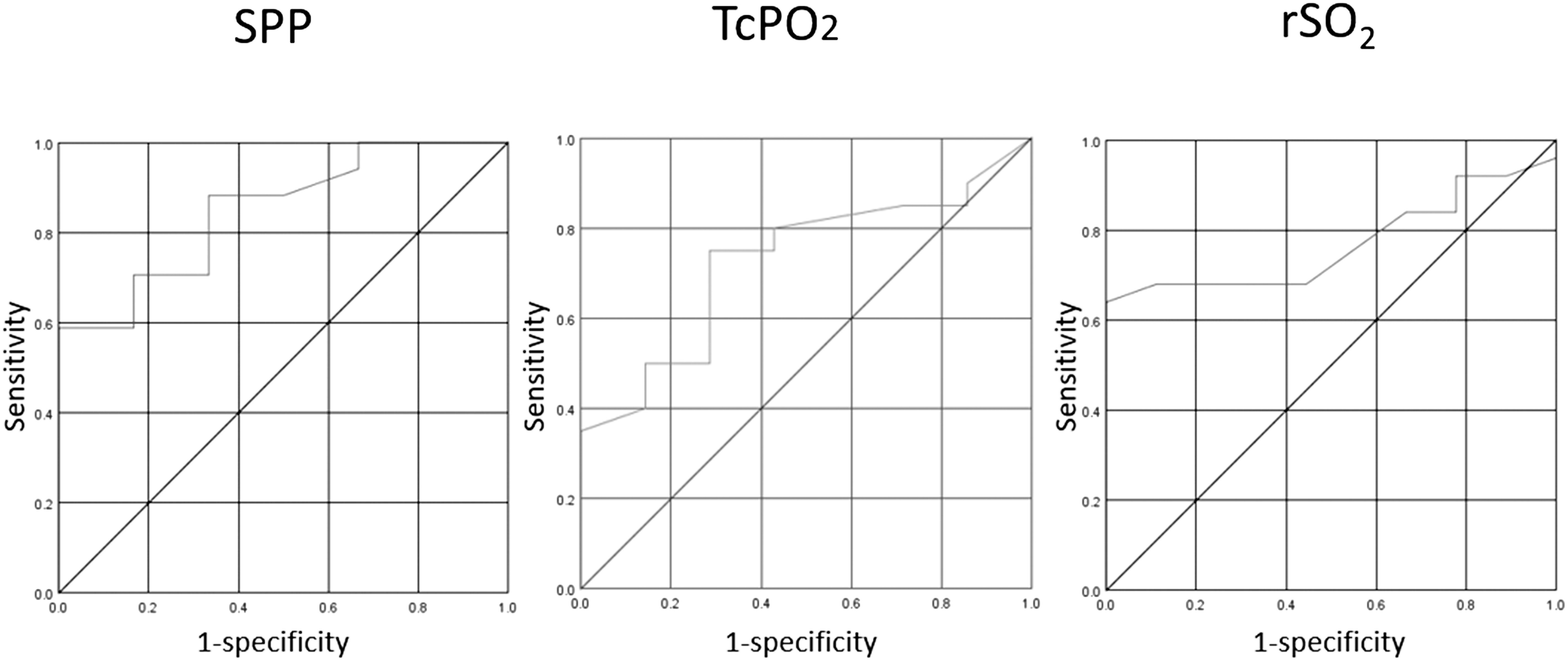
Fig. 4 ROC curves for predicting wound healing. ROC curves for the estimation of cutoff values for predicting wound healing using SPP, TcPO_2_, and rSO_2_.

ROC: receiver operating characteristic; SPP: skin perfusion pressure; TcPO_2_: transcutaneous oxygen pressure; rSO_2_: regional tissue oxygenation saturation

In the ROC analysis of SPP, the area under the curve (AUC) was 0.846 (95% confidence interval [CI] 0.683–1.000, p<0.01). The optimal cutoff value for predicting wound healing was 36.5 mmHg with a sensitivity of 89.5% and a specificity of 71.4%. For TcPO_2_, the AUC was 0.725 (95%CI 0.523–0.927, p=0.081). The optimal cutoff value for predicting wound healing was 10.5 mmHg with a sensitivity of 75.0% and a specificity of 71.4%. ROC analysis of rSO_2_ showed an AUC of 0.780 (95%CI 0.629–0.931, p=0.014). The optimal cutoff value for predicting wound healing was 49.5 mmHg, with 100% sensitivity and 64% specificity (**Fig. 4**).

## Discussion and Conclusion

CLTI occurs in association with chronic ischemia, which often leads to prolonged ulcer healing or necrosis. The ischemia causes tissue hypoxia and deterioration of one’s nutritional status, which hinders wound healing. The appropriate assessment and management of wounds is essential in reducing amputation risk. There has been considerable progress in the field of wound care over the last three decades. Management strategies include debridement, wound dressing, pressure off-loading, and good glycemic control. In our institution, a multidisciplinary approach is adopted before and after EVT to prepare for wound care. In close cooperation with plastic surgeons and dermatologists, effective and efficient EVT should be sought for proper revascularization.

Our results indicate that measuring rSO_2_ is useful for evaluating lower limb blood flow, as are the existing lower limb blood flow tests, such as SPP and TcPO_2_, and also for predicting wound healing after EVT. In this study, EVT was performed in patients with poor limb blood flow and Fontaine stage IV to improve blood flow and heal the ulcers. Overall, the preoperative values indicated poor blood flow with all the methods. After EVT, the blood flow values often improved compared with the preoperative values.

Traditional diagnostic modalities, namely, ABI, SPP, and TcPO_2_, are limited by long measurement times, pain during measurement, measurable skin lesions, or difficulty maintaining the patient’s position during the measurement. In our study, we were unable to measure ABI or SPP in more than 10% of the patients because of intolerable pain during the measurements with a cuff pressure, or to measure TcPO_2_ of the ankle in more than 10% of them because of the difficulty in maintaining a stable patient position during the measurement. Conversely, measuring rSO_2_ using a finger-mounted tissue oximetry device was successful with no difficulty in all ischemic limbs of the participants in this study. Even in patients with rest pain or skin ulcers, the rSO_2_ measurement could be performed at the bedside quickly, simply, painlessly, and repeatedly, in any skin area.

We considered postoperative blood flow evaluations and their relationships with wound healing. SPP, TcPO_2_, and rSO_2_ showed significantly lower values on the first day after EVT in the non-healing group than in the healing group, indicating that wounds are difficult to heal if postoperative values are poor and that measurement of rSO_2_ and existing tests may also reflect this hemodynamic status.

A non-invasive lower limb blood flow evaluation device is important. Pressure measurements using ABI are widely used as objective indicators. For patients with CLTI who had rest pain and those with dialysis and gangrene, the ankle systolic pressures were ≤40 mmHg and ≤60 mmHg, respectively. However, patients with CLTI, including several dialysis patients, often have abnormally high pressure due to calcification, and thus are not accurately evaluated.^20)^ SPP measurements are useful for the diagnosis of CLTI. An SPP value of ≤30 mmHg is a strong predictor of CLTI, with good sensitivity and specificity.^7,21)^ However, SPP examinations require both time and a rest period, and the measurement is often poorly reproducible and varies depending on the patient’s posture. TcPO_2_ values were also used as a predictor of successful wound healing. Reliable wound healing can be expected at values >30 mmHg.^21,22)^ One limitation of this method is its relatively long measuring time. A heating phase of approximately 20 min is required before the measurement can be taken. The resting TcPO_2_ level is deﬁned as the level that changes by less than 2 mmHg in the 2 min following a 10-min response time.^7,23)^ TcPO_2_ cannot be measured intraoperatively and has not been used as an indicator of the endpoint of EVT.

On the basis of these results, we then estimated the cutoff values for wound healing. From the ROC curves, the wound healing cutoff values were 36.5 mmHg for SPP, 11.5 mmHg for TcPO_2_, and 49.5% for rSO_2_. It is generally believed that for wound healing, SPP should be ≥30 mmHg and TcPO_2_ should be ≥40 mmHg.^24,25)^ Although the TcPO_2_ values were slightly different from those reported in the literature, the SPP values obtained in this study were similar to those in the previous studies. The ROC curve of rSO_2_ suggested that rSO_2_>50% is the optimal cutoff value for wound healing with 100% sensitivity for wound healing outcome and should be considered as a guide for treatment. However, in the assessment of SPP for wound prediction, Utsunomiya et al. reported that the optimal cutoff point for predicting wound healing is 30 mmHg, with a sensitivity of 81.4% and a specificity of 69.2%.^25)^ Thus, we believe that rSO_2_ values may be most useful for predicting postoperative wound healing, with values of 50% strongly predicting wound healing.

When focusing on individual cases, we noted that, in several patients, preoperative blood flow was poor and improved after EVT, and wounds were healed. However, some cases had post-EVT values that worsened compared with the preoperative values even with successful EVT procedures. In such cases, the wounds did not heal. Successful EVT does not necessarily improve blood flow and heal ulcers. EVT may sometimes cause damage to blood vessels that are blocking collateral flow and perturbing tissue perfusion. However, there were cases in which both preoperative and postoperative rSO_2_ levels were low, suggesting that we should have planned another revascularization procedure immediately after the initial EVT. At that time, if revascularization is difficult either technically or because of a patient’s general condition, amputation should be considered as a surgical option.

Contrary to conventional devices, the new device may be useful for monitoring tissue oxygenation during EVT, conferring a huge advantage. The device has the potential to determine the need for additional treatment and to help surgeons make decisions during EVT. Targeting rSO_2_ levels in excess of 50% at the ischemic region may become an endpoint of EVT for wound healing.^26)^

Our study has some limitations, in particular because of its single-center, retrospective design and the limited sample size. Moreover, in some cases, ABI, SPP, and TcPO_2_ could not be examined. This particular limitation can, however, be interpreted as an advantage of the new device, which can measure blood flow in more severe cases unsuitable for existing tests. In all cases, the measurement site was limited to the dorsal foot. If a new device that measures rSO_2_ with multiple probes is developed, simultaneous measurement of rSO_2_ at not only the dorsal foot but also the wound site could help improve prediction of wound healing. Finally, only cases of successful EVT without infection were enrolled in this study.

In summary, we evaluated hemodynamic changes after EVT with a new oximeter and compared the device with conventional methods. We identified that the device has advantages of quickness, portability, and less invasiveness in measuring rSO_2_ values at ischemic sites. Retrospective assessment of wound healing indicated that the optimal rSO_2_ value for predicting wound healing is 50%. When performing EVT, rSO_2_>50% may be an endpoint to achieve.
