# Supplementary material for: A Pilot Study Investigating the Use of Regional Oxygen Saturation as a Predictor of Ischemic Wound Healing Outcome after Endovascular Treatment in Patients with Chronic Limb-Threatening Ischemia
Source: Ann Vasc Dis. 2021 Mar 25;14(1):23–30. doi: 10.3400/avd.oa.20-00132 (PMC7991714; doi:10.3400/avd.oa.20-00132)
Supplement: Supplementary Data [file avd-14-1-oa.20-00132_s001.pdf]

Supplementary Table 1: Success rate of blood flow evaluation of the lower limbs

	**All (n = 34)**	**+Wound Healing (n = 25)**	**−Wound Healing (n = 9)**
ABI before EVT	31 (91.2%)	23 (92.0%)	8 (89.9%)
SPP before EVT	24 (70.6%)	18 (72.0%)	6 (66.7%)
$TcPO_2$ before EVT	29 (85.3%)	22 (88.0%)	7 (77.8%)
rSO_2 before EVT	34 (100%)	25 (100%)	9 (100%)
ABI after EVT	29 (85.3%)	22 (88.0%)	7 (77.8%)
SPP after EVT	26 (76.5%)	19 (76.0%)	7 (77.8%)
$TcPO_2$ after EVT	28 (82.4%)	21 (84.0%)	7 (77.8%)
rSO_2 after EVT	34 (100%)	25 (100%)	9 (100%)

ABI, ankle brachial index; EVT, endovascular treatment; SPP, skin perfusion pressure; $TcPO_2$, transcutaneous oxygen pressure; rSO_2, regional tissue oxygenation saturation
